# Simulating selection and evolution at the community level using common garden data

**DOI:** 10.1002/ece3.8696

**Published:** 2022-03-10

**Authors:** Stephen M. Shuster, Arthur R. Keith, Thomas G. Whitham

**Affiliations:** ^1^ Department of Biological Sciences Northern Arizona University Flagstaff Arizona USA; ^2^ Center for Adaptable Western Landscapes Northern Arizona University Flagstaff Arizona USA

**Keywords:** community evolution, community heritability, community selection

## Abstract

A key issue in evolutionary biology is whether selection acting at levels higher than the individual can cause evolutionary change. If it can, then conceptual and empirical studies must consider how selection operates at multiple levels of biological organization. Here, we test the hypothesis that estimates of broad‐sense community heritability, $H_{C}^{2}$, can be used to predict the evolutionary response by community‐level phenotypes when community‐level selection is imposed. Using an approach informed by classic quantitative genetics, we made three predictions. First, when we imposed community‐level selection, we expected a significant change in the average phenotype of arthropod communities associated with individual tree genotypes [we imposed selection by favoring high and low NMDS (nonmetric multidimensional scaling) scores that reflected differences in arthropod species richness, abundance and composition]. Second, we expected $H_{C}^{2}$ to predict the magnitude of the community‐level response. Third, we expected no significant change in average NMDS scores with community‐level selection imposed at random. We tested these hypotheses using three years of common garden data for 102 species comprising the arthropod communities, associated with nine clonally replicated *Populus angustifolia* genotypes. Each of our predictions were met. We conclude that estimates of $H_{C}^{2}$ account for the resemblance among communities sharing common ancestry, the persistence of community composition over time, and the outcome of selection when it occurs at the community level. Our results provide a means for exploring how this process leads to large‐scale community evolutionary change, and they identify the circumstances in which selection may routinely act at the community level.

## Graphical abstract

The evolutionary response to community‐level selection (a) favoring high and low NMDS scores summarizing arthropod communities associated with *Populus angustifolia* between 2004–2006 (after Keith et al., 2010); (b–c) the results of random selection of NMDS scores from the same 2004–2006 NMDS data except that the NMDS scores within these samples were chosen at random without replacement.

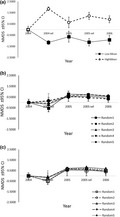

## 1 INTRODUCTION

Heritability and inheritance are often equated, but in fact are different terms. Heritability is statistically defined as the phenotypic covariance for a trait shared among genetically related units (Wade, 2016). Heritability predicts whether selection acting on such units will produce a measurable and lasting evolutionary response (Becker, 1985; Falconer & MacKay, 1996; Lynch & Walsh, 1998). Inheritance, in contrast, is the process by which trait‐causing factors are transmitted across generations (Pierce, 2020). While an understanding of inheritance can be useful for documenting patterns of phenotypic variation, it is not required for estimating heritability in the broad, narrow, or realized sense. Although frequently quantified and therefore increasingly well‐understood at family, group, and population levels (Wade, 2016), heritability as a concept and as a parameter (see Table 1) is poorly known at higher levels of biological organization, particularly among ecological communities (Goodnight, 2000; Shuster et al., 2006; Whitham et al., 2020).

**TABLE 1:** A Glossary of community genetics terminology

Broad‐sense community heritability, $H_{C}^{2}$ – the fraction of the total variation in community phenotype that exists among groups of genetically related plant genotypes (Shuster et al., 2006). This parameter summarizes the phenotypic covariance of arthropod communities that become associated with particular tree genotypes. Estimates of $H_{C}^{2}$ capture the phenotypic covariance among communities related through tree genotype, as well as the evolutionary outcome of selection acting on species within a community context
Community evolution ‐ the outcome of selection operating at multiple levels, resulting in the differential survival and proliferation of communities, which is detectable as a change in the average community phenotype in response to selection (Whitham et al., 2020)
Community heritability – the phenotypic covariance among genetically related communities, either in the broad‐, narrow‐, or realized sense (Shuster et al., 2006; this paper)
Community phenotype – the richness and abundance of individual species found on an individual plant genotype that arise as a result of the traits of individual plant genotypes and interspecific interactions (IIGEs) among community members that colonize or attempt to colonize individual plant genotypes. These assemblages can be quantified using nonmetric multidimensional scaling (NMDS; Whitham et al., 2003)
Community repeatability ‐ the genetic intra‐class correlation of community composition across repeated measurements of individual plant genotypes (Keith et al., 2010)
Community selection differential, *S*_Ci_ – the difference between the average NMDS phenotype of the selected and initial samples of NMDS scores, divided by the standard deviation of the initial sample of scores or *S*_Ci_ = ($Z_{\text{Ci}}^{*}$ – *Z*_Ci_)/*s_Z_*_Ci_ (Eq. 1, this paper)
Community‐level response to selection, *R*_Ci_ – a change in the average community phenotype as a result of community‐level selection, calculated as the difference between the average NMDS scores from trees in the year after selection was imposed, *Z*_Ci+1_, and the average NMDS scores comprising the initial set of communities before selection, *Z*_Ci_, or *R*_Ci_ = *Z*_Ci+1_–*Z*_Ci_ (Eq. 2, this paper)
Community‐level selection – differential survival and/or proliferation of communities often detectable by contextual (multilevel selection) analysis (Goodnight et al., 1992; Whitham et al., 2003)
Foundation species ‐ a single species that defines the structure of a community by creating locally stable conditions for other species; other similar definitions include keystone or dominant species (Ellison et al., 2005)
Heritability ‐ the phenotypic covariance for a shared trait among genetically‐related units (individuals, families, groups, communities; Wade, 2016)
Holobionts – usually a multicellular host and its microbial symbionts (trees and their soil microbes; vertebrates and their gut microbes). Many authors consider the holobiont as a unit of selection in which selection acting on one simultaneously acts on the other (Bordenstein & Theis, 2015)
IIGE – Interspecific indirect genetic effects are interactions by individuals in one species that affect trait expression and fitness among individuals in another species. Note that IIGEs are distinct from indirect genetic effects (IGEs; Moore et al., 1997), which are restricted to interactions among conspecific individuals (Allan et al., 2012; Shuster et al., 2006)
Inheritance – the process by which trait‐causing factors are transmitted across generations (Pierce, 2020)
Narrow‐sense community heritability, $h_{C}^{2}$ – the fraction of the total variation in community phenotype that covaries between parent and offspring host plants, or among host plants that represent half siblings (Smith et al., 2011) specific estimates of $h_{C}^{2}$ will require consideration of breeding design (Falconer & MacKay, 1996)
NMDS – Nonmetric multi‐dimensional scaling is a multivariate statistical procedure generating 1 to n‐dimensional scores summarizing pairwise community dissimilarities based on species abundances on genetically distinct trees generated by the Bray‐Curtis dissimilarity coefficient (Clarke, 1993; Faith et al., 1987; Minchin, 1987)
Realized community heritability, $H_{\text{C(realized[i])}}^{2}$ ‐ the ratio of the response to community‐level selection, *R*_C(i)_, to the community selection differential, *S*_C(i)_ or *R*_C(i)_/*S*_C(i)_ = $H_{\text{C(realized[i])}}^{2}$ (Eq, 3, this paper)
Realized heritability ($h_{\text{realized}}^{2}$) – an estimate of narrow‐sense heritability, *h* ^2^, calculated from a response to selection experiment, in which $h_{\text{realized}}^{2}$ = *R*/*S* (Falconer & MacKay, 1996)
Response to selection (*R*) – a change in the average phenotype as a result of selection, calculated as the difference between the average population phenotype following the selection episode, $Z_{2}^{*}$, and the average population phenotype before selection, *Z*_1_; Falconer & MacKay (1996)
Selection differential (*S*) – the difference between the average phenotype of a selected population, $Z_{1}^{*}$, and the average phenotype of the population before selection, *Z*_1_, divided by the standard deviation of the population before selection, *s*_Z_, or ($Z_{1}^{*}$−*Z*_1_)/*s*_Z_ (Falconer & MacKay, 1996)
Selection in a community context – when natural selection in one species covaries with genetic variation in another species (Shuster et al., 2006)

Why might community heritability matter? How is it related to community selection and community evolution? How do these terms apply to complex ecosystems in nature? Answers to these questions can often be addressed with plants. Many species are perennial enabling repeated measures of their communities. Plants are readily propagated as clones allowing statistical replication, and they can be established in long‐standing common gardens that greatly reduce environmental variation, allowing researchers to focus on the underlying genetic factors. Importantly, many plants represent foundation species because they affect numerous other species and largely define their respective ecosystems (Ellison et al., 2005). In a wide range of foundation plant species, different genotypes support distinct, heritable metacommunities of interacting species, whose species compositions correlate with one another at the individual plant genotype level (e.g., insects, soil microbial decomposers, endophytes, mycorrhizal mutualists, pathogens, lichens; Lamit, Lau, et al., 2015; Lau et al., 2016; Whitham et al., 2012). In such foundation species, selection acting on particular plant genotypes also acts on their associated communities (Gehring, Flores‐Rentería, et al., 2014; Johnson & Gibson, 2021; Whitham et al., 2020). If associated communities were randomly distributed on plants, then selection acting on these communities would have few evolutionary consequences. However, because the communities associated with plants are often specific to plant genotypes, and may involve 100s of species, evolutionary processes operating at the community level are likely to have evolutionary consequences for biodiversity and its conservation as well as for the genetic makeup of plants (Whitham et al., 2020).

The goals of this paper are to: (a) verify the reliability of $H_{C}^{2}$ as a measure for community heritability in the broad sense; we define $H_{C}^{2}$ as the fraction of the total variation in community phenotype that exists among groups of genetically related plant genotypes (Shuster et al., 2006; see Table 1), (b) illustrate how $H_{C}^{2}$ measures the phenotypic covariance of communities on genetically related plants, thereby documenting the outcome of genetic interactions between communities and their host plants (i.e., selection within a community context; Shuster et al., 2006), and (c) demonstrate that estimates of $H_{C}^{2}$ predict the magnitude and direction of the response to community‐level selection. This latter result indicates that while quantitatively resembling estimates of broad‐sense heritability for quantitative traits, *H* ^2^, estimates of community heritability, $H_{C}^{2}$, are analogous in their predictive value to estimates of narrow‐sense heritability, *h* ^2^, when measured for quantitative traits (Becker, 1985; Falconer & MacKay, 1996; Lynch & Walsh, 1998).

We show how community heritability can be measured, and we use common garden data in simulations to evaluate the evolutionary consequences of directional‐ and random selection on associated communities. Our results provide a novel approach for exploring how selection acting on community phenotypes can lead to large‐scale evolutionary change, as well as phenotypic differences that would be overlooked if the focus of investigation was directed only at the individual level. This logic is dependent on the observation that, due to genetic‐based interactions between the plant's traits and the traits of symbiotic species that permit or prevent particular species associations, community members sort themselves out on individual plant genotypes to produce recognizable, repeatable community phenotypes (reviewed in Whitham et al., 2006, 2012).

We summarize the specific methods used for estimating community heritability in the broad‐sense, $H_{C}^{2}$ (Appendix S1). This approach, based on logic developed in Shuster et al. (2006), but not developed into a methods paper, has been used in more than 20 articles to measure the genetic basis underlying community organization (reviews in Whitham et al., 2006, 2008, 2012, 2020). As these studies describe in detail, $H_{C}^{2}$ summarizes the phenotypic covariance of arthropod communities that become associated with particular tree genotypes. This covariance arises because of genetic interactions between trees and local arthropods that cause arthropod species to persist on certain tree genotypes or avoid them. Estimates of $H_{C}^{2}$ capture the phenotypic covariance among communities related through tree genotype, as well as the evolutionary outcome of selection acting on arthropods within a community context; such selection occurs when natural selection in one species covaries with genetic variation in another species (Shuster et al., 2006). In this sense, $H_{C}^{2}$, is distinct from either broad‐sense or narrow‐sense heritability (*H* ^2^ or *h* ^2^, respectively) when measured in the usual quantitative genetic sense (Becker, 1985; Falconer & MacKay, 1996; Lynch & Walsh, 1998).

We next use data from one of these papers (Keith et al., 2010) to illustrate these methods and to test the hypothesis that broad‐sense community heritability, $H_{C}^{2}$, like narrow‐sense heritability for quantitative traits, *h* ^2^, predicts the evolutionary response to community‐level selection. Our results are not merely a recapitulation of the results of these earlier studies. Shuster et al. (2006) emphasized broad‐sense community heritability, $H_{C}^{2}$, as a means for measuring the phenotypic outcome of selection operating within a community context. They presumed that such selection was responsible for the observed similarity of arthropod communities assembling on genetically similar trees, grown in common gardens as well as over broad geographic scales (Bangert et al., 2008). To test this hypothesis, they modeled synthetic communities in which the number, intensity, and fitness consequences of genetic interactions among the species comprising the community were explicitly known. Indeed, their model results showed that empirical estimates of $H_{C}^{2}$ did arise from heritable variation in a tree trait, heritable variation in arthropod traits, and the fitness consequences of the interactions between these traits. Moreover, the intensity of interspecific indirect genetic effects (IIGEs) predicted the empirical value of $H_{C}^{2}$; that is, the degree to which selection within a community context had caused communities to become distinct (Allan et al., 2012).

Keith et al. (2010) showed that the arthropod communities associated with narrowleaf cottonwood (*Populus angustifolia*), are comprised almost entirely of univoltine species, and reassemble each year on replicated tree genotypes in remarkably consistent form. These authors showed that community phenotypes exhibit significant “repeatability” analogous to that observed in standard quantitative traits (Boake et al., 1994). They confirmed that community phenotypes are genetically based and that the interactions between trees and arthropods lead to phenotypic covariance among genetically related communities that persists across years (Whitham et al., 2020).

Here, we use these same, genetic‐based community phenotypes to simulate community‐level selection. In each of three years, as in these previous studies (reviews in Whitham et al., 2012, 2020), we used nonmetric multidimensional scaling (NMDS) scores to summarize community phenotypes. The NMDS scores summarized pairwise community dissimilarities based on species abundances on genetically distinct trees generated by the Bray‐Curtis dissimilarity coefficient (Clarke, 1993; Faith et al., 1987; Minchin, 1987). Although NMDS scores are based on ranks, the univariate scores this analysis produced were continuously variable and thus appropriate for parametric analysis (see also Appendix S1 and below). We then imposed community‐level selection favoring the extremes of these scores and estimated realized community heritabilities from the observed response in community phenotype.

This genetics approach is well established. It mimics the classic quantitative genetics studies to examine selection on high and low oil content in corn (Lambert et al., 2004; Laurie et al., 2004). It has been used to explore the response to individual, group and community selection in a wide range of taxa (Falconer, 1955; Goodnight, 1990a, 1990b; Morris et al., 2005; Singh & Pandey, 1993; Wade, 2016; Wade et al., 1996). Moreover, it is the standard exercise to illustrate and apply the breeder's equation, (*R* = *h* ^2^ *S*, where *R* = the response to selection, *S* = selection differential and $h_{\text{realized}}^{2}$ = realized narrow‐sense trait heritability; Falconer & MacKay, 1996; Pierce, 2020). However, to our knowledge, this is the first use of this approach for quantifying the selection differential, the response to selection, and the realized heritability for community‐level traits. We suggest that this approach will be broadly applicable to all studies of holobionts (e.g., trees and their soil microbes; vertebrates and their gut microbes) in which these symbiotic microorganisms are essential to their survival (Gilbert et al., 2012) and the combined communities are thought to be the primary unit of selection (Bordenstein & Theis, 2015; Roughgarden et al., 2018).

**FIGURE 1:**
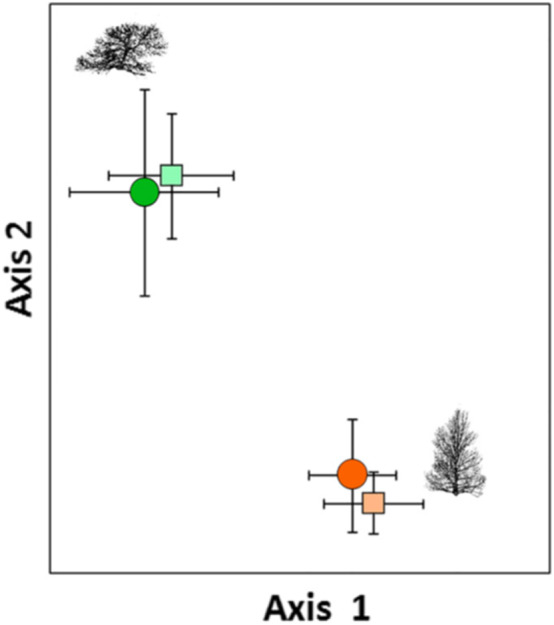
A nonmetric multidimensional scaling (NMDS) ordination of the ectomycorrhizal fungal (EMF) communities on drought‐tolerant and ‐intolerant mature trees of *Pinus edulis* (*large dots*) and seedlings (*small squares*) grown in a common garden. EMF communities differ for each tree drought‐tolerance class (variation in community phenotype), and seedlings acquire the same EMF communities as mature trees in their respective drought‐tolerance class, demonstrating that the community is heritable. On Axis 2, drought selection favoring high NMDS EMF community scores and acting against drought intolerant low NMDS community scores shifts NMDS community scores over time resulting in community evolution. Illustration from Gehring et al. (2017)

What does selection for extremes of NMDS community scores mean in an ecological or evolutionary sense? Do community phenotypes quantified by NMDS scores reflect important community traits such as richness, abundance, composition, and species interactions that if selected upon would result in community evolution? Gehring et al. (2017) provided an example. By comparing the ectomycorrhizal fungal community (EMF) of drought tolerant and drought intolerant mature trees in the wild and their seedlings growing in a common garden (Figure 1), these authors showed (a) that the EMF communities of drought tolerant and intolerant trees are distinct (i.e., they support fungal communities belonging to different systematic divisions), and (b) these communities are heritable in the narrow‐sense in that their seedling progeny support the same EMF communities as mothers. If having the “right” EMF fungal community is important to survive drought, as Gehring et al. (2017) showed, selection acting on trees with extreme NMDS community scores would affect the EMF communities of the next generation and their ability to tolerate drought, which may be crucial with climate change.

Supporting this hypothesis, Gehring, Flores‐Rentería, et al. (2014) showed that over a 16‐year period of drought, selection resulting in differential tree mortality in the wild had shifted the EMF communities of trees to become more drought tolerant. This suggests that selection acting on EMF communities and their host plants can have evolutionary consequences and that community phenotypes quantified by NMDS scores can be a relevant metric for quantifying community evolution. In this example, the authors established that the extremes of NMDS community phenotypes have ecologically recognizable effects.

We emphasize that the procedures we used to generate community‐level selection in this study, did not represent a simulation in the usual sense. We did not attempt to explore a large range of possible outcomes. Our goal was simply to show, using community data available in Keith et al. (2010), what happens when a selected fraction of community phenotypes (e.g., non‐random vs random) are used in each successive year to generate the next year of community phenotypes. We began with a limited number of communities, and in each of three years, reduced the number of communities considered by half, a procedure that severely limited the possible outcomes for our experimental as well as our controlled results. Despite these limitations, our predictions were robust. We predicted a positive evolutionary response to selection for high and low NMDS scores, with realized community heritabilities commensurate in value with measured estimates of broad‐sense community heritability, $H_{C}^{2}$. We also predicted no significant evolutionary response to randomly selected NMDS scores. We define community evolution as the outcome of selection operating at multiple levels, resulting in the differential survival and/or proliferation of communities that is detectable as a change in the average community phenotype (Whitham et al., 2020). We report the results of our experiments and review the applications of this method for understanding community genetics and evolution.

## 2 METHODS

### 2.1 Estimates of broad‐sense community heritability ($H_{C}^{2}$)

Keith et al. (2010) designed an experiment to examine the effect of genotypic variation in plants on arthropod community organization using an 14‐year‐old common garden with replicated clones of nine different *Populus angustifolia* genotypes. All trees planted within the common garden were collected from a single interbreeding population (Martinsen et al., 2001). Trees were identified using molecular markers that allowed exclusion of hybrids and inclusion of genetic variants characteristic of “pure” *P*. *angustifolia*. Genotypes represented in the common garden had been haphazardly selected from trees growing along the Weber River in northern Utah, USA and were planted in a haphazard design. Nine tree genotypes with four to seven replicates each were selected from existing stocks, yielding a total of 44 trees, whose average height was 10–15 m.

Following Wimp et al. (2005), Keith et al. (2010) censused arthropod communities on trees in each of three years (2004–06) and summarized the 102 species (67 families in 12 orders) using nonmetric multidimensional scaling ordination (NMDS). This approach evaluated arthropod community composition as a quantitative trait (Bradshaw & Stettler, 1995), as in studies of diverse multivariate plant traits including phenology, phytochemistry, morphology, sink–source relationships (e.g., Holeski et al., 2012) and interactions with each other that result in different interaction networks for different tree genotypes (e.g., Keith et al., 2017; Lau et al., 2016). Censuses of arthropod community composition for each tree provided individual trait values for this quantitative character according to standard quantitative genetics methods (e.g., in this case, among lineages of clones grown in a common environment; Falconer & MacKay, 1996; Shuster et al., 2006).

### 2.2 Community‐Level selection based on NMDS scores

#### 2.2.1 How NMDS scores describe community phenotype

We assumed that differences in arthropod abundance arose from genetic interactions that had occurred between arthropod genotypes and the genotypes of their tree hosts that either favored their persistence or disfavored the persistence of other individuals or species not found among the sampled arthropod community (c.f., Shuster et al., 2006). This assumption of genetics‐based interactions has been experimentally confirmed for several herbivorous species used in our analyses. For example, transfer experiments showed pronounced differences in resistance among tree genotypes, including arthropod preferences for trees where their survival was greatest and avoidance of tree genotypes where their survival was lowest (e.g., aphids, Whitham et al., 1989; mites, Evans et al., 2008). Experiments have also shown intraspecific genetic differences in mites and aphids in which some genotypes do best on some tree genotypes, but not others (e.g., Evans et al., 2008; Smith et al., 2011). Because some insects, such as aphids can affect 100s of other species including insects, spiders, fungi and birds, their genetics‐based interactions with individual tree genotypes can directly and indirectly affect whole communities of organisms (e.g., Dickson & Whitham, 1996; Keith et al., 2017; Smith et al., 2011) including ecosystem processes such as nutrient cycling (Schweitzer et al., 2005).

Thus, the above and numerous other studies (reviews Whitham et al., 2012, 2020), confirm the use of NMDS scores to quantify a multivariate phenotype arising from the genetic interactions of arthropod symbiont and tree genotypes. For this reason, we expected that directional selection favoring communities expressing particular NMDS scores would change the distribution of NMDS scores in the next year, thereby simulating an evolutionary response to community‐level selection.

#### 2.2.2 Controling for temporal autocorrelation among years

To control for potential autocorrelation of NMDS scores among study years, possibly due to shared maternal environments, position within the common garden, and persistent induced or epigenetic responses among years, we performed a two‐way ANOVA of the NMDS data with tree genotype, year, and their interaction as factors, as well as a Durbin–Watson test for autocorrelation on these data.

#### 2.2.3 Directional community‐level selection on NMDS scores

To simulate community‐level selection based on variation in community phenotype, we selected the NMDS scores with the 10 largest and 10 smallest values from the array of 44 one‐dimensional NMDS scores generated by the 2004 communities within the common gardens described in Keith et al. (2010). This procedure identified two groups of trees whose abundances of arthropod species led to the highest and lowest NMDS ordinations for the 2004 sample. Analogous to the statistical analysis of quantitative traits, this procedure provided no ecological information on the reasons for these community phenotypic similarities or differences, only that they occurred. We consider such ecological anonymity one of the strengths of this approach.

We next identified the 20 trees in the common garden from which these 2004 scores were drawn, isolated these trees and the abundances of the species within these trees’ insect communities from the rest of the 2005 sample, and then performed a one‐dimensional NMDS ordination on these 2005 communities. From this 2005 ordination, we then selected the NMDS scores with the five largest and five smallest values. We identified the 10 trees in the 2006 common garden from which these 2005 scores were drawn, we isolated these trees and the abundances of the species within these trees’ insect communities from the rest of the 2006 sample, and we again performed a one‐dimensional NMDS ordination on these 2006 communities. We tabulated the genotypes and NMDS scores for each episode of selection in Appendix S2.

#### 2.2.4 Random community‐level selection on NMDS scores

Consistent with studies of selection acting on quantitative traits, we expected a random selection of NMDS community scores in each year to produce no evolutionary response, because a random selection of NMDS scores summarizing community phenotypes in each episode of selection would produce no distinguishable change in the average community phenotype. Thus, as a control for our community‐level selection experiment on NMDS scores described above, we performed the same procedures on the 2004–2006 samples of trees and their communities, except with the NMDS scores chosen at random without replacement using a random number generator. We replicated our control procedure 10 times to simulate five independent selection series each on high and low NMDS lineages. We plotted both sets of results.

### 2.3 Measuring the response to community‐level selection

We calculated the mean and 95% confidence limits for the NMDS scores in each of the following samples: (a) the original 2004 sample of scores [2004 initial; *N* = 44 communities], (b) the sample of the 10 high and 10 low scores in the 2004 sample [2004 selected, *N* = 20 communities], (c) the 20 scores of the 2005 communities on the trees identified by the 2004 selected scores [2005 response; *N* = 20 communities], (d) the sample of 5 high and 5 low scores in the 2005 sample [2005 selected; *N* = 10 communities], and (e) the 2006 communities on trees identified by the 2005 selected scores [2006 response; *N* = 10 communities]. We also calculated the mean and 95% confidence limits for each of the five sets of randomly selected NMDS scores used as controls for our high and low NMDS lineages. These methods allowed us to simulate the effects of directional selection on the NMDS‐quantified phenotypes of the arthropod communities inhabiting trees in the common garden, and to compare the results of that selection with randomly selected NMDS scores from the same communities in samples of similar size.

For each i‐th episode of community‐level selection, where i = 2004 or 2005 (no data were available to document a response to selection after 2006 so this episode was not included), we estimated the community phenotypic mean, *Z*_Ci_, and standard deviation, *s_Z_*_Ci_, of the initial distribution of NMDS scores, as well as the mean, $Z_{\text{Ci}}^{*}$, of the distribution of selected NMDS scores from the trees in that selection episode, for our experimental and control communities. We calculated the community selection differential, *S*_Ci_, for each i‐th selection episode as the difference between the average NMDS phenotype of the selected and initial samples of NMDS scores, standardized by the standard deviation of the initial sample of scores, or,

(1) $$S_{\text{Ci}}=\left(Z_{\text{Ci}}^{*}‐Z_{\text{Ci}}\right)/s_{\text{ZCi}}$$

For comparison, we used the tabulated values for selection differentials in Becker, (1985, p. 161–174) based on the number of individuals in each selection episode. We estimated the cumulative selection differential over our two episodes of community‐level selection as the sum of the two selection differentials estimated for the 2004 and 2005 samples. Note that the magnitude of the selection differential depends on the size of the population before selection and the number of selected individuals (Becker, 1985). Because our simulation progressively reduced the numbers of communities included within each episode of community selection, the community selection differentials were expected to become proportionally smaller.

We estimated the response to community‐level selection in each i‐th selection episode, *R*_Ci_, as the difference between the average NMDS scores from trees in the year after selection was imposed, *Z*_Ci+1_, and the average NMDS scores comprising the initial set of communities before selection, *Z*_Ci_, or

(2) $$R_{\text{Ci}}=Z_{\text{Ci}+1}‐Z_{\text{Ci}}$$

Following the breeders’ equation (*R* = *h* ^2^ *S*; Falconer & MacKay, 1996), and methods described in Wade et al. (1996) we estimated the realized community heritability in each i‐th episode of selection (2004, 2005) as the ratio of the response to community‐level selection, *R*_C(i)_, to the community selection differential, *S*_C(i)_ or

(3) $$R_{C\left(i\right)}/S_{C(i)}=H_{C(\text{realized[i}])}^{2}$$

We estimated the realized community heritability overall, $H_{\text{C(realized[total])}}^{2}$, as the ratio of the cumulative response to selection, Σ*R*_C(i)_, to the cumulative community selection differential, Σ*S*_C(i)_, over 2004 and 2005, which we compared with estimates of broad‐sense community heritability, $H_{C}^{2}$, from Keith et al. (2010).

## 3 RESULTS

### 3.1 Visualization of community phenotype

**FIGURE 2:**
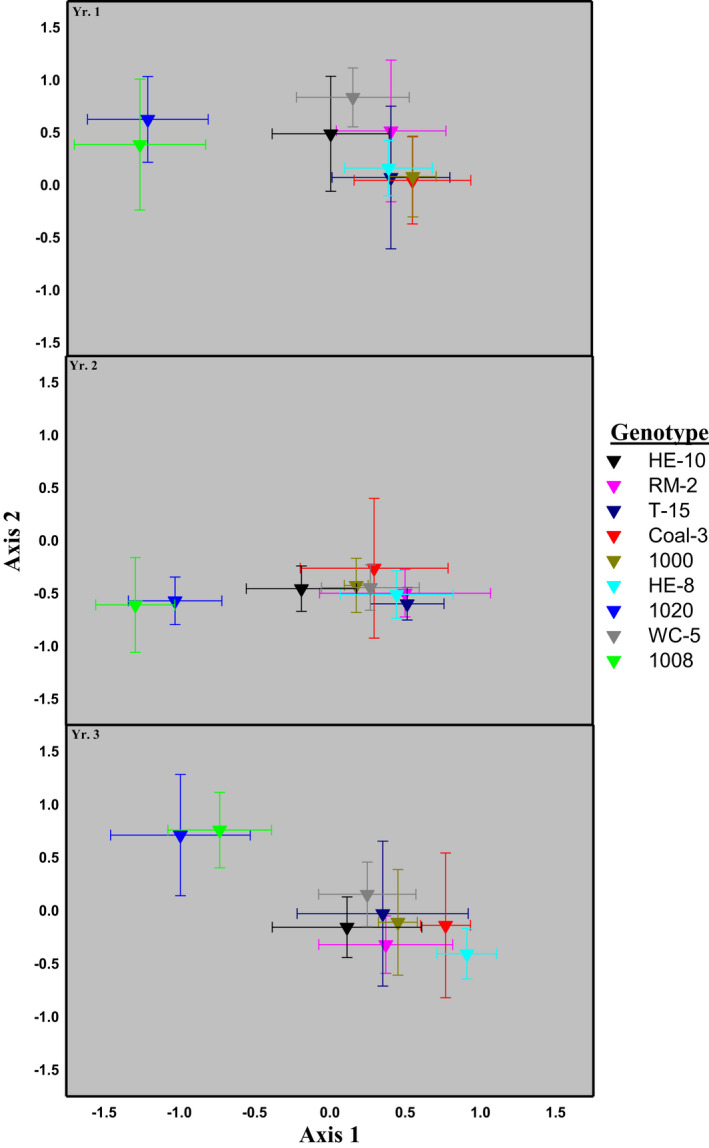
Arthropod data were collected on three consecutive years beginning in 2004 on 44 trees of *Populus angustifolia*, representing clonal replicates of 9 different tree genotypes. Trees were grown for 14 years prior to the study at the Ogden Nature Center in northern Utah. Different tree genotypes supported distinct arthropod communities, shown here as a 2‐D plot of the centroids of NMDS scores calculated for each tree genotype (mean ± 95% CI; data from Keith et al., 2010 who presented histograms rather than centroids). This analysis considered all three years within a single ordination but to aid visualization, each panel shows the position of communities within this analysis for each year. Slight differences among years in the positions of centroids reflect the among‐year and among‐tree genotype variation in arthropod abundances within the common garden

Replicated narrowleaf cottonwood tree genotypes (*N* = 9, 44 total trees) grown in a common garden supported distinct arthropod communities, shown here as a 2‐D plot of the centroids of NMDS scores calculated for each tree genotype (mean ± 95%CI; Figure 2). This analysis considered all three years within a single ordination but to aid visualization, each panel shows the position of communities within this analysis for each year. Slight differences among years in the positions of centroids reflect the among‐year and among‐tree genotype variation in arthropod abundances within the common garden. Despite this variation, note that the positions of centroids relative to one another within a given year, are preserved among the three years of the study. This observational agreement across years is also reflected in repeatability analyses, i.e., the genetic intra‐class correlation of the arthropod community composition across the three years, was high (0.91) indicating a strong, underlying genetic basis, and consistency among years for community phenotype (Boake, 1994). This finding argues against the hypothesis that different vectors of community compositional change were being compared across years. Moreover, if repeatability and heritability of community phenotypes were low or non‐significant (as illustrated in Shuster et al., 2006), a response to community level selection would not have been observed.

Keith et al. (2010) reported significant broad‐sense heritability in community composition for each of the three years of their study [$H_{C}^{2}$ (±95% confidence interval) for year 1 = 0.68 (±0.21); year 2 = 0.68 (±0.21); and year 3 = 0.63 (±0.22)]. Using an alternative restricted maximum‐likelihood analysis, the authors found similar results for $H_{C}^{2}$: year 1 = 0.60 (±0.21); year 2 = 0.66 (±0.23); and year 3 = 0.62 (±0.22) (*p* < .0001). Across all three years, about 65% of the variation in arthropod community composition was related to plant genetic factors.

### 3.2 No evidence of temporal autocorrelation among years

Our two‐way ANOVA to detect temporal autocorrelation in NMDS scores among years was significant overall (*F*_[17,114]_ = 5.83, *p *< .001) and showed significant effects of tree genotype (*F* = 5.17, *p* < .001) and tree genotype‐by‐year interactions (*F* = 7.22, *p *< .001), as is expected if genetic as well as gene‐by‐environmental effects influenced NMDS scores. We found no significant effect of year (*F* = 0.62, *p *= .43). Durbin–Watson's *d* equaled 1.90 with the probability of autocorrelation = 0.033. Values of *d* range from 0–4, with 2.0 indicating no temporal autocorrelation.

### 3.3 Evidence of community‐level selection

**FIGURE 3:**
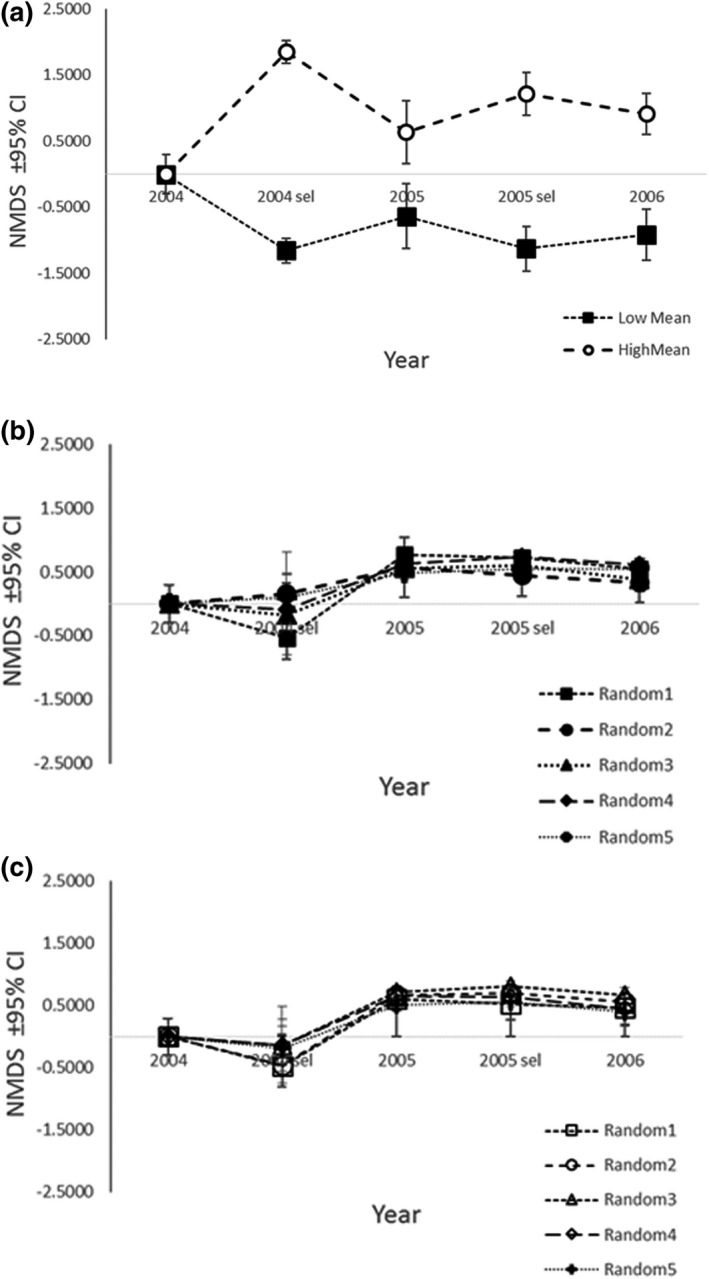
The evolutionary response to community‐level selection favoring high and low NMDS scores using data from Figure 2. NMDS scores summarize arthropod communities associated with *Populus angustifolia* (after Keith et al., 2010). (a) Reading from left to right along the Year axis; 2004 shows that high (dashed line) and low (dotted line) average ± 95% CI NMDS scores were equivalent (*N* = 44). 2004 sel shows the average ± 95% CI NMDS scores for the selected high and low community phenotypes within each lineage in the first episode of community‐level selection (*N* = 10 for each lineage). 2005 shows the response in average ± 95% CI NMDS scores for high and low lineage community phenotypes after one episode of community‐level selection. Note that 95% CI for both lineages are bounded away from zero and from each other (*N* = 10 for each lineage). 2005 sel shows the average ± 95% CI NMDS scores for the selected high and low community phenotypes within each lineage for the second episode of community‐level selection (*N* = 5 for each lineage). 2006 shows the response in average ± 95% CI NMDS scores for high and low lineage community phenotypes after the second episode of community‐level selection. 95% CI for both lineages are bounded away from zero, from each other and from the average NMDS score for 2004 (*N *= 5 for each lineage). (b, c) The results of random selection of NMDS scores from the same 2004–06 NMDS data described above except that the NMDS scores within these samples were chosen at random without replacement using a random number generator. We replicated our control procedure 5 times; (b) “low” lineage; (c) “high” lineage

Our calculated values for community selection differentials on the community phenotypes generated from the 2004 and 2005 were similar to those available in Becker, (1985; Table 2; Figure 3a). Because the selected population was approximately twice as large in 2004 as in 2005, as predicted by Becker, (1985) the values of *S*_C(i)_ and *R*_C(i)_ during the 2004 episode of selection were nearly twice as large in 2005.

**TABLE 2:** Selection differentials (*S*) and responses (*R*) for community‐level selection estimated using Becker, (1985) and calculated using Eqs 10–11; community‐level selection was imposed on NMDS scores generated for arthropod communities associated with 9 genotypes narrowleaf cottonwood (*P*. *angustifolia*; *N* = 44 trees) grown in a common garden since 1990 and sampled between 2004–06

Date	Using Becker (1985)			Using equations 10–11			*N*
	*S*	*R*	$H_{C}^{2}$ realized	*S*	*R*	$H_{C}^{2}$ realized	
2004–05 L	1.30	0.64	0.49	1.16	0.64	0.55	10
2004–05 H	1.30	0.64	0.49	1.85	0.64	0.34	10
2005–06 L	0.74	0.28	0.38	0.63	0.28	0.45	5
2005–06 H	0.74	0.28	0.38	0.76	0.28	0.37	5
Cumulative							
Low	2.04	0.92	0.45	1.79	0.92	0.51	15
High	2.04	0.92	0.45	2.61	0.92	0.35	15
Average			0.45			0.43	

### 3.4 Evidence of community‐level evolution

As expected, if estimates of community heritability predicted the evolutionary response of community‐level selection, our plots of the average community phenotype (±95%CI) over two episodes of community‐level selection (2004, 2005) showed that the average community phenotype, estimated in NMDS scores, diverged in high and in low directions in each selection episode (Figure 3a). The average NMDS score for high and low lineages were significantly different from zero and from each other. Although the average phenotypes continued to diverge, the 95%CI for 2006 NMDS scores within both high and low lineages overlapped with those for 2005 due to the small size (*N* = 5 for each lineage) of the 2006 sample of communities.

### 3.5 Realized community heritability

We estimated the realized community heritability in each i‐th episode of selection (2004, 2005) as the ratio of the response to community‐level selection, *R*_C(i)_, to the community selection differential, *S*_C(i)_ or *R*_C(i)_/*S*_C(i)_ = $H_{\text{C(realized[i])}}^{2}$. Our realized community heritabilities, $H_{\text{C(realized[i])}}^{2}$, estimated using values of *S* from Becker (1985) were 0.49 and 0.38 in the 2004 and 2005 community selection episodes, respectively, with a realized community heritability overall of $H_{\text{C(realized[total])}}^{2}$ = 0.45 for the low and high NMDS lineages. Using our calculated values for community selection differentials, *S*_C(i)_, our values of $H_{\text{C(realized[i])}}^{2}$ were 0.55 and 0.45 for the low NMDS lineages and 0.34 and 0.37 for the high NMDS lineages for the 2004 and 2005 selection episodes, respectively. Our realized community heritability overall, $H_{\text{C(realized[total])}}^{2}$ equaled 0.51 and 0.35 for low and high NMDS lineages, respectively (Table 2). These estimates lacked confidence limits because they were collected from a single common garden. However, the lower boundaries for the estimates of community heritability from Keith et al. (2010) for 2004–06 were 0.47, 0.47, 0.41 (c.f., Shuster et al., 2006) and 0.39, 0.43, 0.40 (c.f., REML), respectively. Our average estimates for realized community heritability using the two methods for estimating *S* above (0.45, Becker, 1985; 0.43, calculated; Table 2) were intermediate between these two sets of values, thus our estimates lie within the boundaries established by these other, statistically rigorous analyses.

### 3.6 Random selection on community phenotype

As expected, if selection on community phenotypes occurred at random within each selection episode, our control replicates of randomly selected “low” and “high” NMDS scores (10 control replicates total) were not distinct from one another (Figure 3b,c). While both positive and negative NMDS scores appeared among the 2004 initial and selected community phenotypes, evidently by chance, the majority of NMDS scores among the community phenotypes in 2005 and 2006 were positive (Figure 3b,c),

## 4 DISCUSSION

### 4.1 Consistency of community phenotypes among years

Keith et al. (2010) found that community phenotypes, identifiable through arthropod species abundances on narrowleaf cottonwood genotypes, were repeatable over three consecutive growing seasons (2004–06). By quantifying the repeatability of this result (*t* = 0.91, *p *< .001; Boake, 1994; Falconer & MacKay, 1996), Keith et al. (2010) confirmed the following two fundamental tenets of community genetics (Whitham et al., 2003, 2006, 2012).

First, over three consecutive years, individual tree genotypes interacted with the phenotypes of associated arthropod species to produce statistically indistinguishable patterns of arthropod abundance, summarized in NMDS scores, which was associated with significant broad‐sense community heritability, $H_{C}^{2}$. This result verifies that intraspecific variation in foundation species’ traits such as phytochemistry, leaf morphology, and bud phenology exerted a powerful influence on the expression of community phenotype (e.g., Bangert, Allan, et al., 2006; Barbour et al., 2009). Such intraspecific plant variation can be so great that it can lead to local adaptation by herbivores to individual tree genotypes (Evans et al., 2008, 2016; Mopper, 1996; Stireman et al., 2005; Wooley et al., 2020).

Second, the consistency of community phenotypes over three seasons indicated that the genetic interactions between tree phenotypes and the genotypes of their associated arthropods are remarkably consistent over time. Individual arthropods generally do not survive between years and new genotypes with compatible phenotypes must reassemble each year (Wimp et al., 2005). Consistent with this result, Zinkgraf et al. (2016) showed that wild cottonwoods preferred by a gall‐forming aphid, *Pemphigus betae*, whose survival approached 100% in 1986, were also the most preferred trees 20 years later in 2006. Similarly, trees avoided in 2006, where aphid survival approached 0%, were also avoided 20 years later (*r* = .80); two candidate genetic markers were associated with the resistance traits of individual trees.

These findings provide repeated tests over multiple years in the same common gardens, of the IIGE hypothesis (Shuster et al., 2006). Specifically, if interactions between tree and arthropod genotypes had no fitness consequences for arthropods settling on cottonwoods, there would be no differences in arthropod community composition within or among cottonwood genotypes planted within the common garden in any given year, much less across multiple study years. The observed association of particular arthropod communities with individual plant genotypes in each of the study years, indicates, not only that selection within a community context had occurred, but also that this genetically based interspecific interaction occurred consistently within each of three seasons.

### 4.2 Selection on communities leading to community evolution

We showed that community‐level selection imposed upon the NMDS scores summarizing the arthropod communities associated with groups of cottonwood tree clones, produced a significant, community‐level, evolutionary response. We consider this response possible because estimates of broad‐sense community heritability, $H_{C}^{2}$, provided a reliable measure of the degree to which particular symbiont species became associated with particular genotypes of host organisms due to genetic‐based interactions between these species (Shuster et al., 2006; Whitham et al., 2008, 2012). Significant community heritability has been demonstrated for disparate communities ranging from ectomycorrhizal fungi, twig endophytes, canopy arthropods, lichens, soil decomposing fungi and bacteria, and leaf pathogens (Lamit, Lau, et al., 2015). These $H_{C}^{2}$ estimates range from 0.18 for lichen community composition on tree trunks (Lamit, Busby, et al., 2015) to 0.70 for the soil microbial community composition beneath individual trees (Schweitzer et al., 2008).

Responses to community‐level selection are not new. Goodnight, (1990a, 1990b, 2000) showed that genetically correlated traits between two *Tribolium* species arose when interspecific interactions occurred, allowing responses to community‐level selection that were not possible through individual selection alone. Goodnight, (1990b) and Goodnight and Craig, (1996) showed that correlated traits between species disappeared when these species were separated from their community context, suggesting that the genetic interactions underlying interspecific relationships were preserved when parent communities gave rise to offspring communities. In microbial communities, Swenson, Wilson et al. (2000), Swenson, Arendt et al. (2000) argued that community heritability arose from the among‐community variance in traits associated with interspecific competitive outcome within communities, providing a genetic explanation for why community‐level selection led to a change in the average community phenotype. Wade, (2016) has suggested that the among‐group component of phenotypic variance provides an estimate of the heritability of group‐related traits, including those expressed at the community level.

A consistent theme in each of these studies is that resemblance in community phenotype arises from genetically based interactions among the constituent community species and thus is the source of community heritability. However, while these studies quantified the responses to community selection, and the among‐community variance in phenotype (group/community heritability), they parameterized neither the selection differentials acting on community phenotypes, *S*_Ci_ (Eq. 1), nor the realized heritabilities of community phenotypes, $H_{\text{C[realized[i]]}}^{2}$ (Eq. 3). We have identified and measured both parameters in this study. We have also measured the community‐phenotypic response to community‐level selection, *R*_Ci_ (Eq. 2) and the broad‐sense community heritability, $H_{C}^{2}$ for these data (Appendix S1). Thus, we have identified and measured each element of a breeders’ equation (c.f., Falconer & MacKay, 1996) for community‐level selection (Eq. 3) in a living experimental system, in this case, *R*_Ci_ = $H_{\text{C[realized[i]]}}^{2}$ *S*_Ci_.

We simulated community‐level selection by including only the most extreme community phenotypes between years. In doing so, we allowed only those genetic interactions between symbionts and the remaining host plants to persist among successive growing seasons, as was shown to occur in the field by Keith et al. (2010). For this reason, we expected and found a community‐level response to our simulation of community‐level selection. However, our control simulations involving random selection showed no such consistency in their responses. Directional selection on heritable community phenotypes produced a significant community‐level response that did not appear when selection on these same communities occurred at random (Figure 3).

Our expectations for these results were grounded by previous experimental studies in the same system showing that with the addition or removal of a single strongly interacting species, the NMDS scores significantly changed (e.g., the addition of a naturally occurring pathogen ‐ Busby et al., 2015; removal of a common aphid ‐ Keith et al., 2017). In another system, Gehring et al. (2014) also found that the NMDS scores of the ectomycorrhizal communities of pinyon pine (*Pinus edulis*) predictably shifted in response to greater interactions with plant parasitism (mistletoe), insect herbivory, and competition with junipers. Importantly, climate change (drought) differentially affected resistance to herbivores to predictably shift community NMDS scores (e.g., Gehring et al., 2017; Stone et al., 2018). In each of these examples, a shift in community NMDS scores was sensitive to and directly associated with interactions with diverse organisms and the abiotic environment.

### 4.3 The importance of common gardens

The strength of interspecific associations, that is, the fidelity with which particular symbiont species and genotypes inhabit particular plant genotypes, is easiest to visualize within a common garden, where symbionts are allowed to associate with replicated plant genotypes. These experimental conditions provide a convenient and intuitive method for measuring such associations. Stronger associations between species lead to higher and more consistent values for $H_{C}^{2}$, a relationship that is borne out in other studies (e.g., communities on *Populus angustifolia* ‐ lichens, $H_{C}^{2}$ = 0.18, Lamit, Busby, et al., 2015; leaf pathogens, $H_{C}^{2}$ = 0.32, Busby et al., 2015; soil microbes, $H_{C}^{2}$ = 0.78, Schweitzer et al., 2008; tri‐trophic interactions of trees, aphids, and avian predators, $H_{C}^{2}$ = 0.80, Bailey et al., 2006). As we have shown here, the value of $H_{C}^{2}$, when estimated directly as well as when inferred by a response to selection, reliably predicts an evolutionary response to community‐level selection. As with any study of quantitative traits, common‐garden estimates will yield the highest estimates for heritability because the environment is standardized. The contingency of such estimates on environmental conditions does not diminish the utility of such measures for predicting evolutionary change.

**FIGURE 4:**
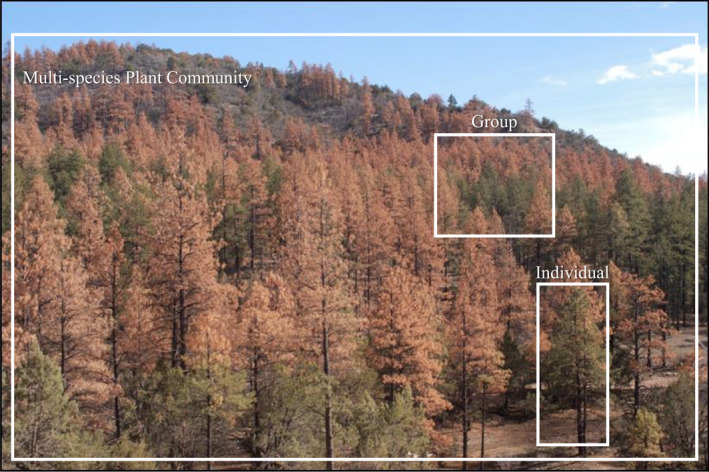
Photo of the ponderosa pine landscape near Prescott, Arizona following the record drought of 2002 when stressed trees were attacked by bark beetles. Boxes show levels of selection in which individual trees and their associated above‐ and belowground communities, groups of adjacent trees and their associated communities, and whole stands of trees, shrubs, grasses and forbes as well as their associated microbial communities died. Numerous studies show that different tree genotypes support different communities and that these communities represent heritable traits in the broad‐ and narrow‐sense (Whitham et al., 2012). Because of community heritability, non‐random tree mortality has the potential to be an evolutionary event for the holobiome of individual trees as well as at higher levels of groups and communities of different plant species with their associated holobiomes (Whitham et al., 2020). Photo by Tom Whitham

While the present study was conducted at the fine scale of communities on individual tree genotypes, generality of our results requires that they scale to higher levels—and they do. For example, in 2002, ponderosa pine (*Pinus ponderosa*) stands and other dominant species in northern Arizona experienced landscape‐level mortality due to record drought (Gitlin et al., 2006; Williams et al., 2020), followed by a bark beetle outbreak that killed stressed trees and their associated holobiont communities at multiple levels. Figure 4 shows how the trees died in a mosaic pattern in which individual trees and their holobiont communities died, groups of trees and their holobiont communities died and communities of trees, shrubs, forbes and grasses and their holobionts were affected. We are aware of no studies that have scaled up beyond the holobiont communities of individual trees, but this photo illustrates how such scaling to higher levels of selection should be possible.

While such mortality may have been random, studies of pinyon pine (*Pinus edulis*) where the underlying genetics were known, established that survivorship was non‐random, had a strong genetic basis (Sthultz et al., 2009) and that, having the “right” ectomycorrhizal communities affected which trees lived or died (Gehring et al., 2017). Furthermore, in a common garden experiment with three interacting species (*Populus fremontii*, *Salix exigua*, *Salix gooddingii*), Grady et al. (2017) showed that the annual net primary productivity was greater for each of the three species when all three were collected from the same site, where they may have adapted to one another, than when they were mixed from different collection sites. This argues that individual species within a community have evolved different IIGEs when associated with different competitors, and that it is possible to scale up from holobiont communities of individual trees and their symbionts to higher levels of complex communities of interacting tree and shrub communities.

In the present study and the examples presented above, selection could have been imposed upon individual tree genotypes, the communities of those genotypes, or both (i.e., multilevel selection; Whitham et al., 2003). Furthermore, mortality was often mosaic; individual dead trees were surrounded by living trees, groups of dead trees were surrounded by living trees, and whole stands lived or died. By quantifying the ectomycorrhizal communities and genetics of individual trees, groups of trees of the same species, and communities of different plant species, selection and evolution may be scaled up to the landscape level. Bangert, Turek, et al. (2006), Allan, et al. (2006), Bangert et al. (2008) found that the genetic basis of arthropod communities on individual trees was scalable from the local to regional levels, but that environmental factors predominated at higher scales. Since no other studies that we are aware of have evaluated the community genetics of groups of trees of the same species or of whole communities of plants across the landscape, researchers know little about genetic scaling at these higher levels, where the role of community genetics may become increasingly important (Bailey et al., 2009; Kagiya et al., 2018; Tovar‐Sánchez et al., 2013).

### 4.4 The predictive power of $H_{C}^{2}$

As explained above, the methods used to quantify broad‐sense community heritability, $H_{C}^{2}$ (Shuster et al., 2006; Whitham et al., 2012) and identify significant variation in community phenotype, are computationally identical. Moreover, because all plant genotypes with their associated communities were represented by multiple clones haphazardly planted in a common garden (Keith et al., 2010), $H_{C}^{2}$, in this study and others, estimates the contribution of all genetic factors influencing community phenotypic variation [$H_{C}^{2}$ = ($\sigma_{\text{among genotype}}^{2}$/$\sigma_{\text{total}}^{2}$); Shuster et al., 2006]. For this reason, we assert that for common garden estimates of $H_{C}^{2}$, the observational components of community resemblance, and the causal components of genetic variance (Falconer & MacKay, 1996) within and among communities are equivalent. This relationship explains the predictive power of $H_{C}^{2}$. Specifically, $H_{C}^{2}$ captures the among‐community fraction of the genetic variance affecting coevolving traits (sensu Goodnight & Craig, 1996; see also Goodnight, 1990a, 1990b, 2000; Swenson, Wilson, et al., 2000; Swenson, Arendt, et al., 2000), and thus identifies the fraction of total genetic variance that influences the composition of ecological communities associated with particular organisms.

However, while genetic interactions are responsible for the phenotypic covariance of genetically related communities, the equivalence of observational and causal components of the genetic factors underlying community heritability, at least in this example, may prevent its partitioning into additive, dominance, and epistatic components (c.f. Becker, 1985; Falconer & MacKay, 1996; Lynch & Walsh, 1998). Other designs may lend themselves more easily to this procedure. Smith et al. (2015) documented evidence of narrow‐sense community heritability, $h_{C}^{2}$, in goldenrod and showed a change in community phenotype with selection acting on individual goldenrods and their associated communities (see also Gehring et al., 2017). Although here, $h_{C}^{2}$ seems analogous to the narrow‐sense heritability of quantitative traits, *h* ^2^, we predict that because offspring are related to each parent by one‐half, genetic interactions with associated species will show less fidelity than is observed with parental clones. Therefore, paradoxically with respect to quantitative genetic predictions, we expect that the response to community‐level selection predicted by $h_{C}^{2}$ will be *less than* that predicted by $H_{C}^{2}$, based on the degree of relatedness considered within the breeding design, a prediction requiring further study.

### 4.5 Comparison of *H* ^2^ and $H_{C}^{2}$

Our results would not be expected if broad‐sense community heritability were strictly analogous to the broad‐sense heritability of quantitative traits. Within the latter parameter, additive as well as non‐additive genetic variance comprise the heritability estimate, and because non‐additive genetic variance tends to erode the response to selection, *H* ^2^ is a poor predictor of the response to selection on quantitative traits.

In contrast, community genetics attempts to correlate the fidelity of the relationship between symbionts and their host plants with the genetic basis underlying that relationship. The consistency of the association among symbionts, measures the degree to which a community trait can be passed from one episode of community‐level selection to the next, and such consistency can be revealed statistically. Indeed, multivariate statistical frameworks such as NMDS capture such associations as univariate scores; scores that summarize the association of multiple symbionts with particular tree genotypes as a community phenotype.

The greater the among‐genotype component of the total variance in community phenotype (i.e., the greater the value of $H_{C}^{2}$), the greater the similarity will be in community phenotype from one episode of community‐level selection to the next (see simulations in Shuster et al., 2006). The relative fidelity of communities to their host organisms is expected to determine not only the relative magnitude of $H_{C}^{2}$, but also the possibility that these communities will respond to community‐level selection. For a given strength of community‐level selection, communities with greater host fidelity are expected to undergo greater evolutionary change.

We suggest that this among‐species fidelity within communities is analogous to additive genetic variance at the population level. Also, as predicted by Shuster et al. (2006), $H_{C}^{2}$ will covary with the heritability of functional plant traits. Moreover, full sibs will share more genes that interact with symbionts and thereby influence the fidelity of this relationship more than half sibs. Because some of these interactions might also arise from dominance or epistasis within the host genome, the rate of decrease will likely only be measurable experimentally. Clearly, the strongest effect of plant genotype on fidelity will be measurable using clones. For this reason, it should be possible to impose selection on communities associated with clones to obtain the clearest response to community‐level selection. Because so many plants reproduce asexually in nature (e.g., Meeus et al., 2007; Schweitzer et al., 2002; Yang & Kim, 2016), we predict that community‐level selection will be common in the wild.

## CONFLICT OF INTEREST

The authors declare no conflicts of interest.

## AUTHOR CONTRIBUTIONS

**Stephen M. Shuster:** Conceptualization (lead); Formal analysis (lead); Funding acquisition (supporting); Methodology (lead); Visualization (equal); Writing – original draft (lead); Writing – review & editing (equal). **Arthur R. Keith:** Formal analysis (supporting); Investigation (lead); Visualization (equal); Writing – review & editing (equal). **Thomas G. Whitham:** Conceptualization (supporting); Funding acquisition (lead); Investigation (supporting); Supervision (lead); Visualization (equal); Writing – original draft (supporting); Writing – review & editing (equal).

## Supporting information

Supplementary Material

Supplementary Material

Supplementary Material

## Data Availability

Data associated with this manuscript are stored in the Dryad Digital Repository (https://doi.org/10.5061/dryad.3bk3j9kmr).
