# Supplementary material for: Simulating selection and evolution at the community level using common garden data
Source: Ecol Evol. 2022 Mar 10;12(3):e8696. doi: 10.1002/ece3.8696 (PMC8928883; doi:10.1002/ece3.8696)
Supplement: Supplementary file 2 — Supplementary Material [file ECE3-12-e8696-s002.docx]

APPENDIX S1.

*ANOVA ON NMDS SCORES*

When community abundance data are replicated on genetically identical host plants obtained through vegetative propagation and the clones grown in a common garden (reviews in Whitham et al., 2003, 2006, 2012), it is possible to measure the among-community component of the phenotypic variance for communities using a one-way ANOVA of the NMDS scores. This procedure provides a univariate measure of the degree to which the genetic interactions that yield fitness effects on community members have produced phenotypically distinct communities. In such analyses, the ANOVA generates a table summarizing the analysis (see Table 1).

*BALANCED EXPERIMENTAL DESIGNS*

When equal numbers of individuals exist within the groups considered in the analysis, for example, a common garden containing 20 tree genotypes, each replicated 5 times (Supporting Information), the analysis is considered balanced. With this design, the variance components within and among the groups of trees on which communities are found are calculated as follows. The variance in community phenotype among groups, σ^2^_G_, equals,

σ^2^_G_  = (MS_G_ - MS_W_)/*k* (1)

where MS_G_ equals the mean squares between groups of genetically identical clones, MS_W_ equals the mean squares within groups of these individuals and *k* equals the number of individuals in each group (Table 1). The variance in community phenotype within groups, σ^2^_W_, equals,

σ^2^_W_ = MS_W_ (2)

where MS_W_ equals the within-group mean squares as defined above. The total variance in community phenotype, σ^2^_total_ = *V*_P_, is the sum of these quantities or,

σ^2^_total_ = σ^2^_G_ + σ^2^_W_. (3)

The variance among groups (Eq. 1) identifies the among-group variance in community phenotype, that is, the variance in community phenotype that appears to be caused by genetic interactions among the genotypes of trees and insects (or other community constituents).

When *k* = n_i_ = the number of individuals per group the design is balanced. The variance within groups (Eq. 2) identifies the variance in phenotype that appears within communities located on genetically identical trees. As in any ANOVA, the larger the value of σ^2^_G_ relative to the total variance (Eq. 3), the more distinct the group means must be.

If the data are organized so that the groups contain trees that are genetically identical, the broad-sense community heritability (*H^2^_C_*), equals the ratio of σ^2^_G_/σ^2^_total_, where σ^2^_total_. = σ^2^_G_ + σ^2^_W_. Thus, for clones with an equal number of communities per clone:

*H^2^_C_*  = σ^2^_G_/σ^2^_total_ (4)

Note that this value equals the intraclass correlation, *t*, that is, the fraction of the total phenotypic variance that exists *among groups* (Becker, 1985; Falconer & McKay, 1989; Lynch & Walsh, 1998). The larger this fraction is, the more distinct the communities are. This relationship was illustrated on a landscape scale by Bangert et al., (2006a, b, 2008), who showed that spatial scales spanning over 800 km, arthropod community phenotype covaries with genetic distance among host trees. Moreover, when species richness was plotted as a function of spatial scale, as in the familiar species–area curve, this curve was also a function of genetic diversity among host trees, suggesting that these relationships have an underlying genetic basis.

When the data consist of the abundances of species that are associated with clones of host organisms, *t* equals *H^2^_C_* because clones are genetically identical and the estimate of *H^2^_C_* summarizes all of the genetic factors (including the genetic interactions between trees and other organisms) that produce variation in community phenotype. If the communities on trees are related to each other as siblings through a single parent, then this ratio must be multiplied by 2 (because the groups are only related by ½ and therefore only share one half of the additive genetic variance underlying community traits). If the hosts supporting communities represent half sibs, *t* must be multiplied by 4.

These latter considerations are the basis for estimates of narrow-sense community heritability because, depending on the breeding design of the host organism array (i.e., the causal components of genetic variance), it becomes possible to generate estimates of community heritability in the narrow-sense, *h*^2^_C,_ based on how communities resemble one another (i.e., the observational components of genetic variance; c.f., Smith et al., 2015). How *h*^2^_C,_ relates to community phenotype and more details on how it can be estimated will be addressed elsewhere.

*UNBALANCED EXPERIMENTAL DESIGNS*

When the numbers of individuals within the groups considered in the analysis are unequal, the analysis is considered unbalanced, and a correction is necessary, both for the calculation of the sums of squares and for the confidence limits estimates above. Many statistical packages automatically calculate this correction term (CT) when they print out values for mean squares. However, to calculate variance components correctly, it is still necessary to adjust the value of *k*, the number individuals per group (Becker, 1985). The adjusted value of *k*, is represented as *k*_1_, whose value is,

*k*_1_ = 1/(G-1) [n. – (Σn_i_^2^ / n.)]. (5)

For example, if the experimental designed consisted of the following group sizes A-E: A= 8, B = 4, C = 5, D = 6 and E = 8, the value of *k*_1_ would equal,

*k*_1_ = 1/(5-1) [31 – (8^2^ + 4^2^ + 5^2^ + 6^2^ + 8^2^) / 31] = 6.10. (6)

The variance components among-groups (communities) and within groups are calculated as shown in Eqs. 1-4 with the following exception; the variance in community phenotype among groups, σ^2^_G_, for an unbalanced experimental design equals,

σ^2^_G_  = (MS_G_ - MS_W_)/*k*_1_ (7)

where *k*_1_ equals the adjusted value of *k* shown in Eq. 7. Appendix B summarizes methods for generating confidence limits for balanced and unbalanced experimental designs. Additional applications of this approach using REML and Permanova procedures are available in (Keith et al., 2010, 2017; Smith et al., 2015).

*CONFIDENCE LIMITS FOR A BALANCED EXPERIMENTAL DESIGN*

The parameters needed for calculating standard errors for *H^2^_C_* include *t*, the intraclass correlation, G = the number of clones, and *k* = number of individuals per clone. For a balanced experimental design, the standard error of *H^2^_C_* equals,

S.E. = { [ 2(1- *t*)^2^ (1 + (*k* -1) *t* )^2^ ] / [*k* (*k* - 1)(G - 1)] }^1/2^ (B1)

Multiplying this value by 1.96 generates 95% confidence limits (Sokal & Rohlf, 1995), which can be subtracted from the estimate of *H^2^_C_* to give the lower boundary for this estimate. If that boundary is greater than zero, the estimate of heritability is statistically significant.

*CONFIDENCE LIMITS FOR AN UNBALANCED EXPERIMENTAL DESIGN*

Becker, (1985) provides an adjusted formula for estimating the standard error of heritability estimates calculated with unbalanced designs with the assumption that *t* is normally distributed, an assumption that is more likely to be met with larger sample sizes. Here the standard error equals,

S.E. = {[2(n. – 1)(1- *t*)^2^ (1 + (*k*_1_ -1) *t* )^2^] / [*k*_1_^2^ (n. - G)(G - 1)]}^1/2^ (B2)

As described above, multiplying this value by 1.96 generates 95% confidence limits that can be added and subtracted from estimates of *H^2^_C_*. The estimate is significant if the lower boundary is greater than zero.

Sokal, R. R., & Rohlf, J. F. (1995). Biometry, 3rd ed. W. H. Freeman.

**Table S1.** The sources of phenotypic variation in the phenotypes of communities inhabiting genetically identical clones of host organisms.

Source of variation df SS MS F EMS

Between (clones, families, etc.) S-1 SS_S_ MS_S_ MS_S_/MS_W_ σ^2^_W_ + *k*(σ^2^_S_)

Individuals within (clones, etc) n.-S SS_W_ MS_W_ σ^2^_W_

___ ___

Total df_total_ SS_total_

In this table, S = number of groups of genetically identical clones, although families or other groups of individuals of known genetic relationship may be used; n_i_ = number of individuals in the i-th group (i.e., per clone, family, etc.)*; k* = n_i_ in expected mean squares (EMS) = n_i_ if the design is balanced, = k_1_ if design is unbalanced; n. = total number of individuals in the analysis; df = degrees of freedom; SS = sum of squares; MS = mean square = SS/df; EMS = expected mean square, i.e., the variance components that are summarized by the MS.
